# Development and internal validation of a clinical prediction model for osteopenia in Chinese middle-aged and elderly men: a prospective cohort study

**DOI:** 10.1186/s12891-024-07526-7

**Published:** 2024-05-20

**Authors:** Ting Li, Jing Zeng, Zimo Pan, Fan Hu, Xiaoyan Cai, Xinjiang Wang, Guanzhong Liu, Xinghe Hu, Xinli Deng, Meiliang Gong, Xue Yang, Yanping Gong, Nan Li, Chunlin Li

**Affiliations:** 1 Department of Endocrinology, the Second Medical Centre & National Clinical Research Centre for Geriatric Disease, Chinese PLA General Hospital, Beijing, China; 2 Department of Nephrology, the Second Medical Centre & National Clinical Research Centre for Geriatric Disease, Chinese PLA General Hospital, Beijing, China; 3 Department of Radiology, the Second Medical Centre & National Clinical Research Centre for Geriatric Disease, Chinese PLA General Hospital, Beijing, China; 4 Department of Clinical Laboratory, the Second Medical Centre & National Clinical Research Centre for Geriatric Disease, Chinese PLA General Hospital, Beijing, China; 5 Department of Outpatient, the Second Medical Centre & National Clinical Research Centre for Geriatric Disease, Chinese PLA General Hospital, Beijing, China

**Keywords:** Osteopenia, Nomogram, Risk factors, Cohort study, Male

## Abstract

**Background:**

Early identification of patients at risk of osteopenia is an essential step in reducing the population at risk for fractures. We aimed to develop and validate a prediction model for osteopenia in Chinese middle-aged and elderly men that provides individualized risk estimates.

**Methods:**

In this prospective cohort study, 1109 patients who attend regular physical examinations in the Second Medical Centre of Chinese PLA General Hospital were enrolled from 2015.03 to 2015.09. The baseline risk factors included dietary habits, exercise habits, medical histories and medication records. Osteopenia during follow-up were collected from Electronic Health Records (EHRs) and telephone interviews. Internal validation was conducted using bootstrapping to correct the optimism. The independent sample T-test analysis, Mann_Whitney U test, Chi-Square Test and multivariable Cox regression analysis were utilized to identify predictive factors for osteopenia in Chinese middle-aged and elderly men. A nomogram based on the seven variables was built for clinical use. Concordance index (C-index), receiver operating characteristic curve (ROC), decision curve analysis (DCA) and calibration curve were used to evaluate the efficiency of the nomogram.

**Results:**

The risk factors included in the prediction model were bone mineral density at left femoral neck (LNBMD), hemoglobin (Hb), serum albumin (ALB), postprandial blood glucose (PBG), fatty liver disease (FLD), smoking and tea consumption. The C-index for the risk nomogram was 0.773 in the prediction model, which presented good refinement. The AUC of the risk nomogram at different time points ranged from 0.785 to 0.817, exhibiting good predictive ability and performance. In addition, the DCA showed that the nomogram had a good clinical application value. The nomogram calibration curve indicated that the prediction model was consistent.

**Conclusions:**

Our study provides a novel nomogram and a web calculator that can effectively predict the 7-year incidence risk of osteopenia in Chinese middle-aged and elderly men. It is convenient for clinicians to prevent fragility fractures in the male population.

## Background

According to a working group of the World Health Organization (WHO), osteopenia(low bone mass, LBM) is defined as a T score that is higher than − 2.5 but less than − 1.0 [1]. With the rapid growth of aging population worldwide, osteopenia has become a major public health problem because of high prevalence and serious health hazard in the elderly. The National Osteoporosis Foundation (NOF) estimates that 10.2 million Americans have osteoporosis (OP) and that an additional 43.4 million have LBM [2]. Meanwhile, in China, the overall prevalence rate of OP in people over 50 years old is 19.2%, and the prevalence rate in men is 6.0%; the overall prevalence rate in people with osteopenia who need prevention and treatment is 46.4%, and in men, it is as high as 46.9% [3]. Although the risk of fracture is greater among patients with OP than among those with osteopenia, the much larger number of persons with osteopenia means that this group represents a substantial portion of the population at risk for fracture [4]. OP and fragility fractures associated with LBM pose a huge economic burden and health risk to the whole society. A report regarding the economic burden of OP in the European Union estimated the costs of incident and prior fragility fractures at €37 billion, with an expected increase by 25% in 2025 [5]. Moreover, active prevention of osteoporotic fracture can be beneficial to prolong life expectancy and improve quality of life for the elderly [6].

There are many well-known OP or fracture risk assessment tools (International Osteoporosis Foundation (IOF), fracture risk assessment tool (FRAX®) and Qfracture score) were available by far [7]. However, prediction models for osteopenia are almost absent. Therefore, it is necessary to establish a prediction model of LBM based on the need for the advanced diagnosis and treatment of chronic diseases. The current techniques used for diagnosis of OP and fracture risk models developed for prediction of fracture risk based on risk factors are primarily for postmenopausal women, and similar effective measurement tools for men are not available [8–10]. However, the incidence of LBM or OP in elderly men is not low. In addition, osteoporotic fractures in men may have more serious consequences, higher morbidity and mortality than those in women [11, 12].

Therefore, we believe that it is important to develop predictive models of LBM in men. The goal of this study was to develop and validate a clinical prediction model to estimate the risk of osteopenia in Chinese middle-aged and elderly men.

## Methods

### Study participants

**Fig. 1:**
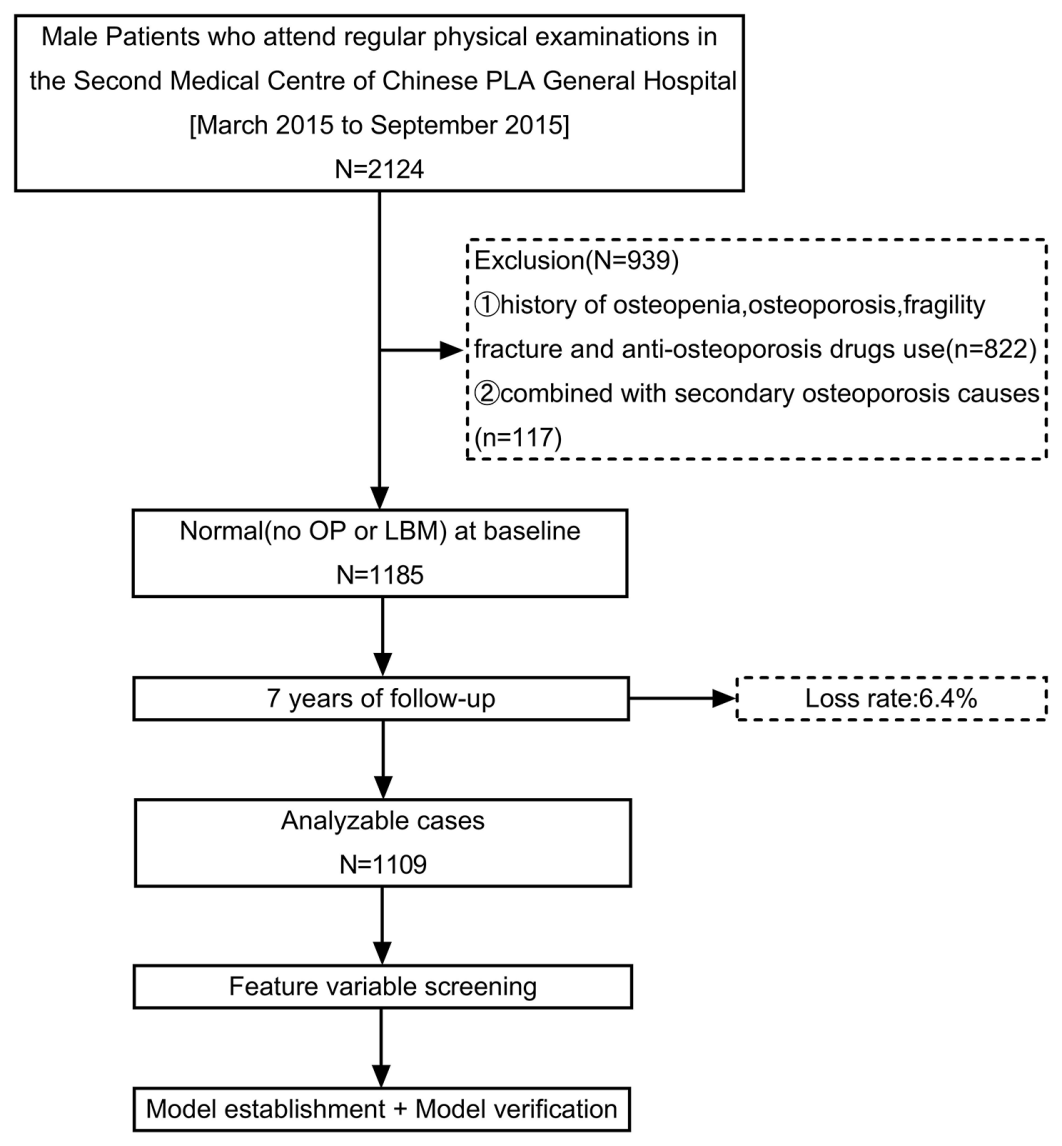
Flow diagram of study design LBM, low bone mass; OP, osteoporosis

Patients of this prospective study were enrolled during the period between March 2015 and September 2015, from the Second Medical Centre of Chinese PLA General Hospital. All enrolled patients had comprehensive physical examination results and had a definite outcome of either osteopenia or not. This study was approved by the Ethics Committee of Chinese PLA General Hospital (ID: S2021-094-01). The inclusion criteria were as follows: (i)patients with normal bone mineral density (BMD) that were measured by dual-energy X-ray absorptiometry (DXA); (ii)age ≥ 45 years old; (iii)Chinese male patients. Meanwhile, the exclusion criteria were as follows: (i)patients with history of osteopenia, OP and fragility fracture and anti-osteoporosis drugs use; (ii)patients combined with secondary OP. Finally, 1185 non-OP patients at the baseline were included for a 7-year non-interventional follow-up. The follow-up period was from March 2015 to September 2022. The follow-up time for each patient was calculated from baseline to diagnosis of LBM or the end of follow-up period in months. Ultimately, 1109 patients completed the second survey and were included in the study. The follow-up response rate was 93.6%, and the detailed research flow chart is shown in Fig. 1.

### Data collection

On one side, the standardized self-administered questionnaires pertaining to personal history (histories of smoking, drinking, coffee, carbonated beverage and tea consumption), dietary habits (such as staple food, egg, red meat, white meat, dairy products, soy products) and exercise habits (exercise frequency, exercise duration, exercise intensity) were conducted by trained residents. We inquired about smoking, drinking, coffee, carbonated beverage and tea consumption as ‘never’ and ‘past or current’. On the other side, any medical or fracture histories (such as hypertension, dyslipidemia, diabetes, coronary heart disease (CHD), cerebrovascular disease (CVD), chronic kidney disease (CKD), fatty liver disease (FLD), benign prostatic hyperplasia (BPH), cataract) and medication records (such as antihypertensive drugs, oral hypoglycemic drugs, insulin, statins, acid inhibitors, sleeping pills) were collected in detail from electronic medical records of these patients. In order to minimize sampling bias, data were obtained by communicating effectively with medical workers and double checking with them.

The height, weight, waist circumference (WC), respiratory rate (RR), heart rate (HR), and blood pressure (BP) were measured by uniformly trained investigators. The subjects wore thin shirts and stood upright on the bottom plate of a stadiometer to measure their height and weight. WC was measured at the thinnest part of the waist (the horizontal circumference of the waist through the umbilical point). body mass index (BMI) was then calculated by weight (kg)/height (m^2^). The BP, HR and RR were measured after the subjects rested for 10 min. An electronic sphygmomanometer (Omron) was used to measure BP three times, and the average value was taken as the data analysis.

Biochemical measurements were performed using standardized methods in the central laboratory of our institution. Blood samples with fasting for more than 8 h were extracted to detect for white blood cell count, red blood cell count, platelet count, hemoglobin (Hb), serum calcium, serum phosphorus, serum magnesium, fasting blood glucose (FBG), hemoglobin A1c (HbA1c), serum albumin (ALB), alanine aminotransferase (ALT), aspartate aminotransferase (AST), gamma-glutamyltransferase (GGT), serum creatinine (Cr), blood urea nitrogen (BUN), triglyceride (TG), total cholesterol (TC), high density lipoprotein cholesterol (HDL), low density lipoprotein cholesterol (LDL), prothrombin time (PT), activate part plasma prothrombin time (APTT), prothrombin time (TT), fibrinogen (FIB), thyroid stimulating hormone (TSH), total triiodothyronine (TT3), total thyroxine (TT4), free triiodothyronine (FT3), free thyroxine (FT4), luteinizing hormone (LH), follicle-stimulating hormone (FSH), testosterone (T), estradiol (E2), progesterone (P), osteocalcin (OST), type I procollagen amino-terminal peptide (P1NP), β isomer of C-terminal telopeptide of type I collagen (β-CTX), parathyroid hormone (PTH), 25-hydroxy-vitamin D [25(OH)D] and alkaline phosphatase (ALP). The same day, blood samples with breakfast after 2 h were extracted to detect for postprandial blood glucose (PBG).

BMD scores were obtained from completed DXA scans by chart abstraction for each patient. At our hospital, we use a GE Lunar DXA (GE Healthcare, Madison, WI, USA). According to the diagnostic criteria from the WHO, the definition of normal is T-score ≥–1, the definition of osteopenia is − 2.5 < T-score <–1, and the definition for OP is T-score ≤ − 2.5.

### Statistical analysis

Continuous variables were expressed as mean ± standard deviation or median (interquartile range, IQR), while categorical variables were presented as frequencies (percentages, %). To compare the difference between groups, we use chi-squared test for categorical variables, T-test analysis and Mann_Whitney U test for continuous variables. Variables found to be associated with LBM at a *p* value <0.05 were then entered into multivariable Cox proportional hazard model identify significantly the predictive factors associated with osteopenia. Both proportional hazards assumption and schoenfeld residuals were used to prove that it conforms to the Cox model requires that the ratio of the hazards of the occurrence of the outcome for any 2 individuals remains constant during the entire follow-up. Subsequently, factors with prognostic significance in the multivariable Cox regression analysis were utilized to build a prediction model for osteopenia in Chinese middle-old men and a nomogram was used to visualize the model. Internal validation was conducted using bootstrapping to correct the optimism. Areas under the receiver operating characteristic curve (ROC) curve (AUC), and decision curve analysis (DCA) were used to assess discrimination of the model, while the calibration plot was used to graphically evaluate the calibration of the nomogram in our prospective cohorts. All analyses were conducted using R software (version 4.3.0), and *p* values less than 0.05 were considered statistically significant in each statistical analysis.

## Results

### Characteristics of the study cohort

This was a 7-year retrospective cohort study. We collected data from 1109 patients from the Second Medical Centre of Chinese PLA General Hospital between March 2015 and September 2015. After a follow-up time of 7 years, 451 participants had an osteopenia, with an overall incidence rate of 40.67%. Those with LBM were more likely to be older male, with lower BMI, lower PINP, lower BMD at left femoral neck (LNBMD), lower Hb, lower ALB, lower PROG, history of smoke, history of tea consumption, FLD, cataract, assisted walking, higher PBG and higher TT3(Table 1).

**Table 1:** Comparison of characteristic variables between LBM group and non-LBM group

Variables	Total(*n* = 1109)	LBM(*n* = 451)	non-LBM(*n* = 658)	*p* value
Age(years)	65(59,75)	66(60,74)	65(59,75)	0.291
**BMI(kg/m**^**2**^)	24.96(23.42,27.06)	24.36(22.86,26.15)	25.18(23.46,27.01)	**<0.001**
Waistline(cm)	92.00(87.80,98.00)	92.00(87.00,97.00)	92.00(88.00,98.00)	0.283
RR(b.p.m)	18.00(17.00,18.00)	18.00(17.00,18.00)	18.00(17.00,18.00)	0.825
HR(b.p.m)	70.00(65.00,77.00)	70.00(65.00,78.00)	70.00(65.00,76.00)	0.066
SBP(mmHg)	125.00(120.00,134.00)	125.00(120.00,134.00)	126.00(119.00,135.00)	0.800
DBP(mmHg)	75.00(70.00,80.00)	75.00(70.00,80.00)	75.00(70.00,80.00)	0.410
WBC(10e12/L)	5.77(4.82,6.68)	5.77(4.89,6.63)	5.78(4.81,6.72)	0.954
**N(%)**	0.578 ± 0.079	0.583 ± 0.079	0.575 ± 0.079	**0.095**
**Hb(g/L)**	150.00(143.00,157.00)	148.00(141.00,155.00)	152.00(144.00,159.00)	**<0.001**
**ALB(g/L)**	46.39 ± 2.68	45.16 ± 2.39	46.24 ± 2.53	**0.000**
BUN(mmol/L)	5.60(4.80,6.60)	5.50(4.70,6.50)	5.60(4.80,6.70)	0.210
**Cr(µmol/L)**	85.00(77.00,94.00)	84.00(76.00,92.00)	86.00(78.00,95.00)	**0.004**
TC(mmol/L)	4.26(3.69,4.81)	4.30(3.70,4.87)	4.22(3.68,4.76)	0.399
TG(mmol/L)	1.20(0.93,1.69)	1.20(0.92,1.59)	1.21(0.93,1.72)	0.404
HDL(mmol/L)	1.25(1.07,1.46)	1.27(1.09,1.48)	1.23(1.06,1.45)	0.064
LDL(mmol/L)	2.70(2.17,3.26)	2.73(2.15,3.30)	2.69(2.19,3.21)	0.702
**LDH(U/L)**	171.00(154.00,189.00)	169.00(152.00,187.00)	172.00(156.00,191.00)	**0.042**
CK(U/L)	108.40(82.70,149.30)	105.10(80.80,144.90)	110.00(84.18,152.33)	0.060
CK_MB(U/L)	12.30(10.60,14.60)	12.30(10.40,14.50)	12.30(10.70,14.60)	0.585
GGT(U/L)	22.00(17.00,30.50)	21.00(16.00,30.00)	23.00(17.00,31.00)	0.095
ALP(U/L)	60.00(51.00,70.00)	60.00(51.00,70.00)	60.00(51.00,70.25)	0.997
AMY(U/L)	67.00(55.00,84.00)	67.00(55.00,85.00)	67.00(54.00,83.00)	0.661
HbA1c(%)	5.70(5.40,6.00)	5.70(5.50,6.10)	5.70(5.40,6.00)	0.300
FBG(mmol/L)	5.56(5.22,6.07)	5.58(5.21,6.11)	5.55(5.22,6.04)	0.840
**PBG(mmol/L)**	8.25(6.96,10.01)	8.88(7.61,10.95)	7.77(6.55,9.45)	**0.000**
TT4(nmol/L)	98.97 ± 15.93	99.66 ± 16.01	98.50 ± 15.87	0.233
**TT3(nmol/L)**	1.58(1.43,1.74)	1.60(1.44,1.76)	1.57(1.42,1.72)	**0.033**
FT3(pmol/L)	4.71(4.40,5.04)	4.71(4.41,5.05)	4.73(4.38,5.03)	0.699
FT4(pmol/L)	15.96(14.67,17.37)	15.89(14.59,17.22)	15.98(14.72,17.44)	0.302
TSH(µIU/mL)	2.06(1.47,2.86)	2.11(1.50,2.81)	2.04(1.46,2.92)	0.939
OCN(ng/ml)	15.01(11.70,18.63)	14.75(11.35,18.55)	15.26(11.89,18.65)	0.248
PTH(pg/ml)	37.90(30.19,48.76)	37.71(29.59,49.21)	38.16(30.59,48.64)	0.409
**PINP(ng/ml)**	32.87(24.03,42.79)	31.80(23.48,41.32)	33.61(24.56,43.90)	**0.037**
β-CTX(ng/ml)	0.28(0.18,0.39)	0.28(0.18,0.38)	0.29(0.18,0.40)	0.292
25(OH)D (ng/ml)	21.63(15.72,27.69)	21.96(16.59,27.44)	21.42(15.37,27.76)	0.380
TS(ng/ml)	4.51(3.40,5.78)	4.55(3.35,5.88)	4.51(3.42,5.71)	0.836
E2(pmol/L)	86.93(60.30,114.70)	85.29(59.53,112.45)	87.76(60.84,116.69)	0.369
LH(mIU/ mL)	7.15(4.58,10.77)	6.90(4.42,10.24)	7.32(4.76,11.12)	0.107
FSH(mIU/ mL)	11.73(6.63,19.28)	11.21(6.59,18.32)	11.98(6.64,20.62)	0.417
PRL(µg/L)	16.12(9.33,23.02)	15.22(9.15,22.05)	16.54(9.46,24.05)	0.220
**PROG(nmol/L)**	1.20(0.64,1.92)	1.12(0.61,1.81)	1.25(0.65,1.98)	**0.039**
**LNBMD(g/cm**^**2**^)	0.97(0.90,1.04)	0.92(0.87,1.00)	1.00(0.93,1.07)	**0.000**
**LHBMD(g/cm**^**2**^)	1.06(0.99,1.14)	1.02(0.96,1.10)	1.08(1.01,1.16)	**0.000**
**smoking**				
No	627(56.5%)	186(41.2%)	441(67.0%)	**<0.001**
Yes	482(43.5%)	265(58.8%)	217(33.0%)	
**tea consumption**				
No	350(31.6%)	84(18.6%)	266(40.4%)	**<0.001**
Yes	759(68.4%)	367(81.4%)	392(59.6%)	
milk				
No	362(32.6%)	136(30.2%)	226(34.3%)	0.144
Yes	747(67.4%)	315(69.8%)	432(65.7%)	
**staple food**				
No	860(77.5%)	374(82.9%)	486(73.9%)	**0.000**
Yes	249(22.5%)	77(17.1%)	172(26.1%)	
fruit				
No	258(23.3%)	97(21.5%)	161(24.5%)	0.252
Yes	851(76.7%)	354(78.5%)	497(75.5%)	
meat				
No	783(70.6%)	319(70.7%)	464(70.5%)	0.938
Yes	326(29.4%)	132(29.3%)	194(29.5%)	
egg				
No	616(55.5%)	246(54.5%)	370(56.2%)	0.579
Yes	493(44.5%)	205(45.5%)	288(43.8%)	
**assisted walking**				
No	1067(96.2%)	427(94.7%)	640(97.3%)	**0.027**
Yes	42(3.8%)	24(5.3%)	18(2.7%)	
calcium supplement				
No	712(64.2%)	294(65.2%)	418(63.5%)	0.570
Yes	397(35.8%)	157(34.8%)	240(36.5%)	
**vitamin D supplement**				
No	696(62.8%)	300(66.5%)	396(60.2%)	**0.032**
Yes	413(37.2%)	151(33.5%)	262(39.8%)	
**exercise**				
No	300(27.1%)	96(21.3%)	204(31.0%)	**0.000**
Yes	809(72.9%)	355(78.7%)	454(69.0%)	
fall				
No	1046(94.3%)	432(95.8%)	614(93.3%)	0.080
Yes	63(5.7%)	19(4.2%)	44(6.7%)	
diabetes				
No	786(70.9%)	316(70.1%)	470(71.4%)	0.624
Yes	323(29.1%)	135(29.9%)	188(28.6%)	
hypertension				
No	475(42.8%)	188(41.7%)	287(43.6%)	0.523
Yes	634(57.2%)	263(58.3%)	371(56.4%)	
dyslipidemia				
No	326(29.4%)	127(28.2%)	199(30.2%)	0.454
Yes	783(70.6%)	324(71.8%)	459(69.8%)	
CHD				
No	811(73.1%)	340(75.4%)	471(71.6%)	0.160
Yes	298(26.9%)	111(24.6%)	187(28.4%)	
**CVD**				
No	968(87.3%)	410(90.9%)	558(84.8%)	**0.003**
Yes	141(12.7%)	41(9.1%)	100(15.2%)	
CKD				
No	1074(96.8%)	437(96.9%)	637(96.8%)	0.935
Yes	35(3.2%)	14(3.1%)	21(3.2%)	
**FLD**				
No	799(72.0%)	288(63.9%)	511(77.7%)	**<0.001**
Yes	310(28.0%)	163(36.1%)	147(22.3%)	
BPH				
No	620(55.9%)	237(52.5%)	383(58.2%)	0.062
Yes	489(44.1%)	214(47.5%)	275(41.8%)	
**cataract**				
No	1021(92.1%)	406(90.0%)	615(93.5%)	**0.037**
Yes	88(7.9%)	45(10.0%)	43(6.5%)	
PD				
No	1107(99.8%)	449(99.6%)	658(100.0%)	0.087
Yes	2(0.2%)	2(0.4%)	0(0%)	

Continuous variables were expressed as mean ± SD or median (interquartile range). Categorical variables are expressed as frequencies (percentages )

*p* < 0.05 (two-sided) was considered statistically significant

Bold face indicates statistical significance

25(OH)D, 25-hydroxy vitamin D; ALB, albumin; ALP, alkaline phosphatase; AMY, amylase; β-CTX, β isomer of C-terminal telopeptide of type I collagen; BMI, body mass index; BPH, benign prostate hyperplasia; BUN, blood urea nitrogen; CHD, coronary heart disease; CK, creatine kinase; CK_MB, creatine kinase isoenzyme MB; CKD, chronic kidney disease; Cr, serum creatinine; CVD, cerebrovascular disease; DBP, diastolic blood pressure; E2,estradiol; FPG, fasting plasma glucose; FLD, fatty liver disease; FSH, follicle-stimulating hormone; FT3,free triiodothyronine; FT4,free thyroxine; GGT, gamma-glutamyl transpeptidase; Hb, hemoglobin; HbA1c, hemoglobin A1c; HDL, high-density lipoprotein cholesterol; HR, heart rate; LBM, low bone mass; LDH, lactate dehydrogenase; LDL, low-density lipoprotein cholesterol; LH, luteinizing hormone; LHBMD, bone mineral density of left total hip; LNBMD, bone mineral density of left femoral neck; N, neutrophil; OCN, osteocalcin; PBG, postprandial blood glucose; PD, Parkinson’s disease; PINP, serum carboxy-terminal propeptide of type I collagen; PRL, prolactin; PROG, progesterone; PTH, parathyroid hormone; RR, respiratory rate; SBP, systolic blood pressure; TC, total cholesterol; TG, triglyceride; TS, testosterone; TSH, thyroid stimulating hormone; TT3,total triiodothyronine; TT4,total thyroxine; WBC, white blood cells

### Model development and web-based calculator

**Fig. 2:**
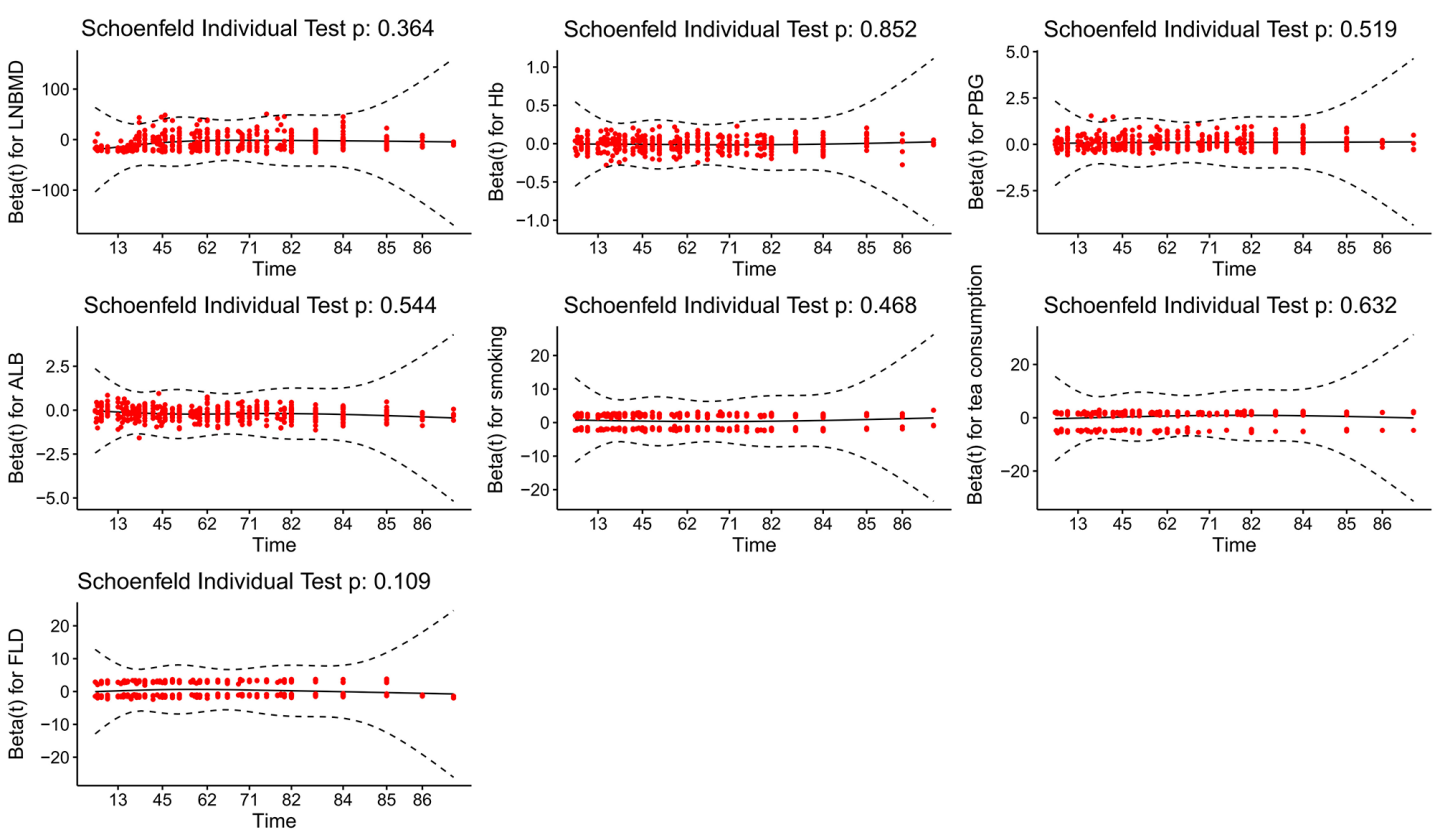
Schoenfeld residuals ALB, albumin; β, partial regression coefficient; Hb, hemoglobin; HR hazard ratio, CI confidential interval, FLD, fatty liver disease; LNBMD, bone mineral density of left femoral neck; PBG, postprandial blood glucose

**Fig. 3:**
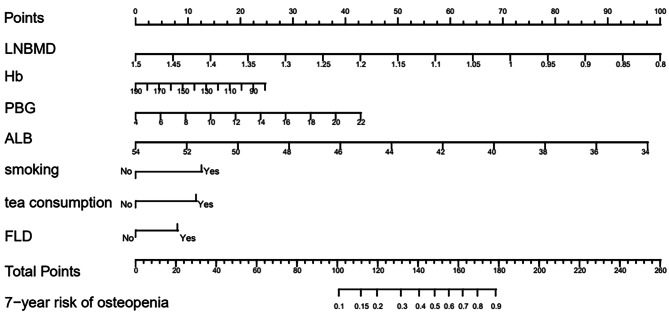
Nomogram prediction for the risk of osteopenia Predictors contained in the prediction nomogram included LNBMD, Hb, 2hPG, ALB, smoke, tea, FLD. ALB, albumin; β, partial regression coefficient; Hb, hemoglobin; HR hazard ratio, CI confidential interval, FLD, fatty liver disease; LNBMD, bone mineral density of left femoral neck; PBG, postprandial blood glucose

To screen risk factors for osteopenia, we performed univariate analysis of patients with and without osteopenia, as shown in Table 1. Then we included all variables with *p* < 0.05 in the univariate analysis into the multivariable COX regression analysis. Results of the multivariate Cox regression analysis are shown in Table 2; and verify that all variables are consistent with the Proportional Hazards assumption (Table 3; Fig. 2). The above multivariable COX regression analysis (Table 2) suggests that the prediction model finally identifies seven predictors, including LNBMD, Hb, PBG, ALB, smoking, tea consumption and FLD. Low LNBMD (HR, 0.00345; 95% CI, 0.00097–0.01224; *p* < 0.001), low Hb level (HR, 0.99113; 95% CI, 0.98289–0.99945; *p* = 0.037), higher PBG level (HR, 1.09922; 95% CI, 1.06192–1.13783; *p* < 0.001), low ALB concentration (HR, 0.82394; 95% CI, 0.79446–0.85452; *p* < 0.001), smoking (HR, 1.64669; 95% CI, 1.36069–1.99281; *p* < 0.001), tea consumption (HR, 1.58007; 95% CI, 1.24279–2.00888; *p* < 0.001), FLD (HR, 1.37232; 95% CI, 1.12944–1.66744; *p* = 0.001) were significantly associated with increased risk of osteopenia in the model. The fitted regression risk model was then rendered as a nomogram that can effectively predict the 7-year incidence risk of osteopenia in Chinese middle-aged and elderly men (Fig. 3). An example interpretation of this nomogram is as follows: a male patient with LNBMD = 1.00 g/cm^2^, Hb = 120 g/L, PBG = 12mmol/L, ALB = 46 g/L, smoking, never drinking tea, and a history of FLD. The seven features attract the scores of 71.5,16,19,39,12.5,0 and 8, respectively (total 166). The nomogram indicates that the risk of an osteopenia is almost 76%.

**Table 2:** Categorical Predictors of Osteopenia Risk(Cox regression model)

Variables	β	HR(95%CI)	*p*-value
LNBMD	-5.66991	0.00345(0.00097–0.01224)	< 0.001
Hb	-0.00891	0.99113(0.98289–0.99945)	0.037
PBG	0.09460	1.09922(1.06192–1.13783)	< 0.001
ALB	-0.19366	0.82394(0.79446–0.85452)	< 0.001
smoking	0.49877	1.64669(1.36069–1.99281)	< 0.001
tea consumption	0.45747	1.58007(1.24279–2.00888)	< 0.001
FLD	0.31650	1.37232(1.12944–1.66744)	0.001

Adjusted for age, BMI, neutrophil, lactate dehydrogenase, total triiodothyronine, serum carboxy-terminal propeptide of type I collagen, progesterone, staple food, assisted walking, vitamin D supplement, exercise, cerebrovascular disease and cataract

*p* < 0.05 (two-sided) was considered statistically significant

ALB, albumin; β, partial regression coefficient; Hb, hemoglobin; HR hazard ratio, CI confidential interval, FLD, fatty liver disease; LNBMD, bone mineral density of left femoral neck; PBG, postprandial blood glucose

**Table 3:** Proportional hazards assumption

variables	chisq	df	*p*-value
LNBMD	0.824	1	0.364
Hb	0.035	1	0.852
PBG	0.415	1	0.519
albumin	0.368	1	0.544
smoking	0.526	1	0.468
tea consumption	0.229	1	0.632
FLD	2.569	1	0.109

*p* > 0.05 (two-sided) was considered to meet the proportional hazards assumption

ALB, albumin; Hb, hemoglobin; HR hazard ratio, CI confidential interval, FLD, fatty liver disease; LNBMD, bone mineral density of left femoral neck; PBG, postprandial blood glucose

**Fig. 4:**
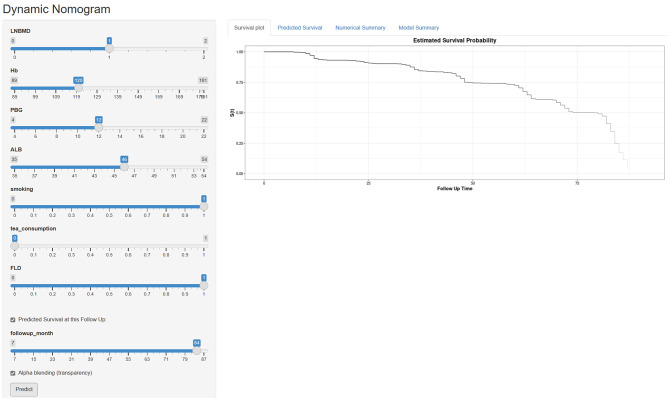
The online web-based calculatora for predicting osteopenia among Chinese middle-old male patients a: https://fafa717.shinyapps.io/DynNomapp/

An online web-based calculator based on our predictive model was developed to allow clinicians to enter the values of the 7 variables required for the risk score with automatic calculation of the likelihood (with 95% CIs) that a male patient will develop osteopenia (https://fafa717.shinyapps.io/DynNomapp/) (Fig. 4).

### Model validation

**Fig. 5:**
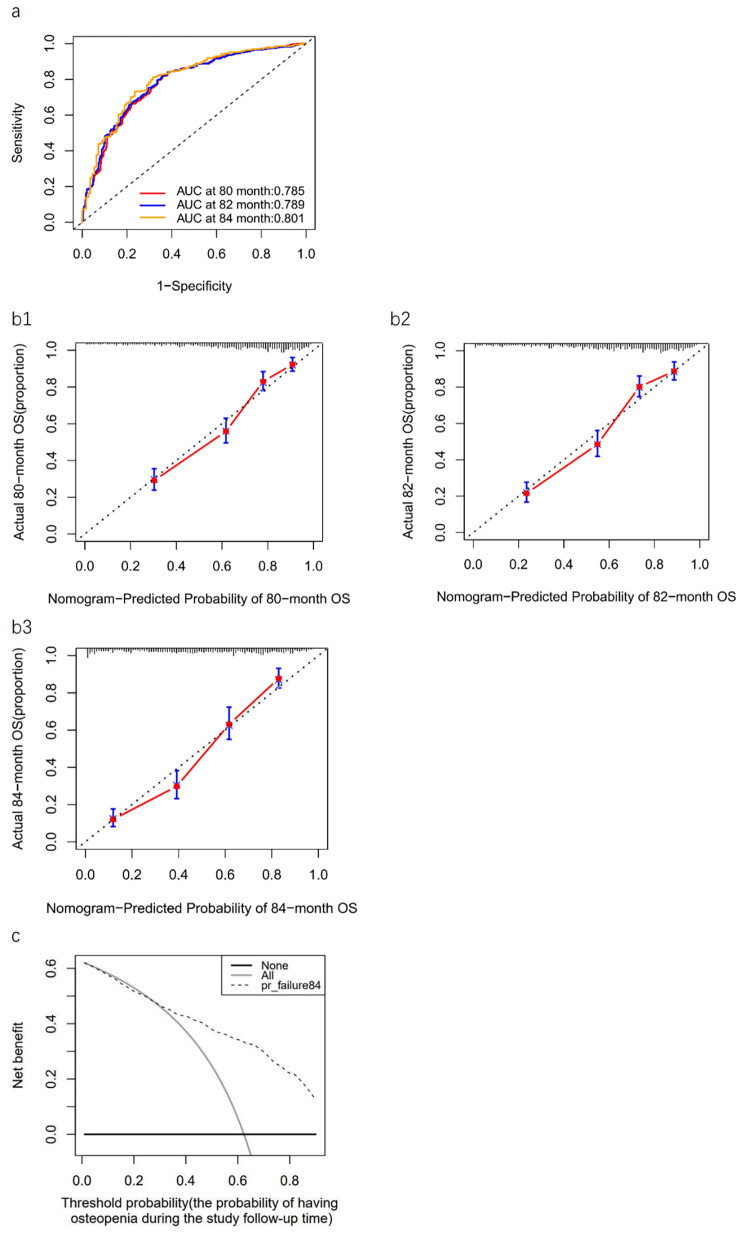
Receiver operating characteristic curve, clinical decision curve analysis, and calibration curves (**a**) ROC curve of the predictive osteopenia risk nomogram. The y-axis represents the sensitivity of the risk prediction, the x-axis represents the 1-specificity of the risk prediction. The red line represents the performance of the nomogram at 80 month. The blue line represents the performance of the nomogram at 82 month. The orange line represents the performance of the nomogram at 84 month. (b1-b3) Calibration curve of the predictive osteopenia risk nomogram. The y-axis represents actual diagnosed cases of osteopenia, the x-axis represents the predicted risk of osteopenia. The diagonal dotted line represents a perfect prediction by an ideal model, the solid line represents the predictive power of the actual model, with the results indicating that a closer fit to the diagonal dotted line represents a better prediction. (**c**) DCA curve of the predictive osteopenia risk nomogram at 84 month. The y-axis represents the net benefit. The thick solid line represents the assumption that no patients have osteopenia, the thin solid line represents the assumption that all patients have osteopenia, the dotted line represents the osteopenia risk nomogram.ROC, receiver operating characteristic; DCA, decision curve analysis

On the one hand, the Concordance index (C-index) for the prediction model in the cohort was 0.773 (95% CI, 0.772–0.786; Likelihood ratio test: *p* < 0.001) and was 0.762 by bootstrapping validation (95% CI, 0.759–0.766), indicating that the model had good refinement. The AUC of the risk nomogram were 0.785 at the 80th month, 0.789 at the 82th month and 0.801 at the 84th month(Fig. 5a), which suggested that the model had adequate predictive capabilities and great discrimination. On the other hand, the calibration curve for the risk nomogram at different points in time exhibited good calibration in our prospective cohort (Fig. 5b1-3). Furthermore, the threshold probability was defined as the probability of having osteopenia during the study follow-up time (at 84-month) and Fig. 5c shows the clinical DCA for the risk nomogram at the 84th month. In DCA, the curve for our model showed a positive net benefit for the threshold probabilities between 23% and 90% compared to the strategies of assuming that all or none of the patients had osteopenia. The DCA shows that using this risk nomogram to predict the 7-year risk for osteopenia was beneficial in clinical work.

## Discussion

Based on the characteristics of OP and osteoporotic fracture, early prevention is particularly more important than treatment [13]. Therefore, a simple and accurate tool to identify the risk of osteopenia is very important in developing countries such as the People’s Republic of China. Current tools for OP or fracture prediction suffer from a number of major weaknesses. A study reported poor results when attempting to validate the use of Osteoporosis Self-assessment Tool for Asians (OSTA) for identifying postmenopausal OP in a Chinese cohort as diagnosed with lumbar spine DXA BMD measurements [14]. Meanwhile, due to regional differences, lack of appropriate cohort data, and design flaws, some studies have found that FRAX® has limitations in clinical use [15]. The study of Harvey et al. [16]showed that the accuracy of using FRAX® to predict fracture risk in men may be lower than in women. A possible explanation of the relative underestimation of fractures by FRAX® could be its design, which adjusts fracture risk based on expected mortality rates, resulting in a decline of absolute risk for those aged over 80 years [17]. Therefore, we believe that it is very meaningful clinical work to study the prediction of LBM in elderly men. In this study, we developed a predictive model applicable for middle-aged and elderly men to help guide the strategy of LBM. Seven predictors were finally selected as the most appropriate features to build this model: LNBMD, Hb, ALB, PBG, FLD, smoking and tea consumption.

### Baseline BMD

The BMD measurements play an important role in assessing bone mass and predicting the risk of fractures. Moreover, we found that BMD at any site was still associated with the risk of fracture in women who did not fracture over the first decade after the BMD measurement [18]. Melton et al. showed in 225 postmenopausal women followed up for a median of 16 years that femoral neck BMD predicted the risk of osteoporotic fracture as well in the first 10 years of follow-up as in the subsequent 10 years [19]. Bone loss rates are variable [20], but because bone loss proceeds at a rate of only a few percent per year, evidence shows that baseline BMD would still predict a sufficiently large proportion of the variation in BMD after several years to be clinically useful for deriving intervention thresholds [21]. However, many osteoporosis prediction models do not include this index, which may be because most baseline BMD data is not readily available in clinical work. Whereas, we have detailed BMD data in our database so that we can use BMD at baseline to predict osteopenia. At the same time, considering that degenerative changes in the elderly, aortic calcification or other spinal morphological abnormalities may affect the DXA detection of lumbar BMD, the LNBMD detected by DXA was selected as the study variable. Finally, our study found that LNBMD at baseline was an important protective factor for osteopenia in middle-aged and elderly men. Our results are also generally consistent with studies performed outside of China regarding associations between baseline BMD and osteoporotic fracture risk. Moreover, we innovatively incorporated the baseline BMD in the clinical predition model.

### Nutritional status (Hb/ALB)

Nutritional status are strongly associated with BMD. Hb and ALB levels are important indicators of patients’ nutritional status. Recently, different studies indicated that a low Hb level was associated with a high risk of OP in the adult population [22–25]. On the one hand, relative hypoxia caused by decreased Hb may increase the differentiation and activity of osteoclasts by increasing the expression of hypoxia-inducible factors and osteoclast-specific factors and the formation of extracellular acidic environment, resulting in increased bone resorption [24, 26]. On the other hand, the effects of hypoxia on osteoblasts, including inhibition of proliferation and differentiation and alteration of bone mineralization, suppress bone formation [27]. Meanwhile, other studies came to the conclusion that low ALB concentration was significantly and independently associated with low BMD [28, 29]. In fact, hypoalbuminemia activates osteoclasts and inhibits osteoblasts through nuclear factor kappaB (NFκB) factors and other infammatory cytokines [30]. Moreover,

hypoalbumine mia causes a decrease in insulin-like growth factor-1 synthesis, thereby leading to a decreased number of osteoblasts, decreased cellular activity, increased osteo clast lifespan, increased bone resorption, and decreased bone remodeling [31]. Overall, in our model, we similarly found that the low Hb level and low ALB concentration were risk factors for osteopenia.

### Glycolipid metabolism and FLD

Abnormal glycolipid metabolism is another risk factor that may be associated with low BMD. In a previous study, similar to diabetics, high prediabetics have lower trabecular bone score (TBS) than normoglycemic individuals [32]. And another study revealed the significant causal effect of HbA1c on BMD [33]. In insulin resistant states, insulin signaling leads to expansion of bone marrow adipose tissue and decreased trabecular BMD [34]. Recent reports indicated that the chronic inflammatory microenvironment induced by hyperglycaemia activates the NLR Family Pyrin Domain Containing 3 (Nlrp3) inflammasome and promotes the production of inflammatory factors, leading to the inhibition of proliferation and differentiation of osteoblasts [35]. Meanwhile, exposure to high glucose inhibits nuclear factor erythroid 2­related factor 2 (Nrf2)/ Kelch­Like ECH­Associated Protein 1 (Keap1) signalling, leading to oxidative stress induction, assembly of Nlrp3, activation of Caspase1, and subsequent initiation of pyroptosis.This cascade culminates in disrupted bone remodelling and exacerbated osteoporosis [36]. In this study, we discovered that after adjusting for age, BMI, and other confounding factors, Chinese middle-aged and elderly men with high PBG levels have a higher risk for osteopenia. Therefore, actively controlling blood sugar has a positive effect on preventing osteopenia.

Moreover, nonalcoholic fatty liver disease (NAFLD) is now widely recognized as a highly prevalent metabolic disease. Multiple studies suggested that NAFLD was associated with decreased BMD and an increased risk of OP or osteoporotic fractures [37–39]. In the circumstance of liver inflammation and fibrosis, hepatic stellate cells are activated and release oncofetal fibronectin, which acts on osteoblasts to decrease bone formation [40]. Furthermore, liver also secretes peripheral colony-stimulating factor-1 (CSF1) and inflammatory cytokine-like tumor necrosis factor (TNF) which respectively bind the colony-stimulating factor-1 receptor (c-Fms) and TNF receptor on osteoclast precursors, thereby increasing bone resorption [41]. Our prospective cohort study in China showed that middle-aged and elderly men with FLD were at a higher risk of osteopenia than non-FLD men.

### Smoking

It is widely known that smoking is a risk factor for bone loss and plays a key role in osteopenia. Studies showed that being a smoker was associated with the prevalence of OP or osteopenia compared with being a nonsmoker and that there was a strong nonlinear positive dose-response relationship between serum cotinine levels and OP and osteopenia [42, 43]. Our result are consistent with the above findings. At present, the mechanism through which smoke causes bone loss is becoming clearer. A study found that cigarette smoke exposure enhanced bone remodeling stimulated by mechanical force and increased osteoclast numbers [44]. Also, cigarette smoke extract increased the number of osteoclasts by inhibiting osteoclast apoptosis via the mitochondrial reactive oxygen species/cytochrome C/caspase 3 pathway [44]. Other findings suggest that smoke exposure induces RANKL activation-mediated by NFκB, which could be a “smoke sensor” for bone remodeling [45].

### Tea consumption

There is still no consistent conclusion about the effect of tea consumption on osteopenia. Some studies suggesting a potential link between tea consumption and reduced BMD or increased fracture risk. Caffeine, a regular part of tea, has also been suggested to affect bone through derangement of calcium metabolism, alteration of vitamin D responses, and other mechanisms [46]. Whereas, other studies found that tea consumption was linked to a lower risk of OP, particularly among women and middle-aged people [47, 48]. Catechin, the main polyphenols found in green tea with potent anti-oxidant and anti-inflammatory properties, can enhance osteoblastogenesis by enhancing osteogenic differentiation of mesenchymal stem cells (MSCs) and increasing osteoblastic survival, proliferation, differentiation, and mineralization [49]. In addition, a two-sample Mendelian randomization study discovered that there was no statistical power to confirm a causal relationship between tea consumption and the risk of OP [50]. However, in this study, we found that tea consumption was a risk factor for osteopenia. And we believe that differences in methodology, selected populations, and duration/timing of the studies may account for study outcome discrepancies. Since, a large-scale, placebo-controlled, long-term randomized trial with a tea regimen intervention of optimal duration is required to determine its efficacy on osteopenia.

In summary, our study has several strengths. First, to our knowledge, this is the first prospective study that addressed LBM prediction in a study cohort that included Chinese middle-aged and elderly men. Second, the included population in this study attend regular physical examinations in the Second Medical Centre of Chinese PLA General Hospital, so the follow-up population was highly stable. Third, as the seven predictors in our model were both accessible at a common enough physical examination, the early prevention of osteopenia-related factors may reduce the risk of OP and osteoporotic fracture. Fourth, in middle-aged and elderly men, this model could screen patients with a high risk of osteopenia and help clinicians to make correct health guiding decisions for each patient.

### Limitations

However, there are several shortcomings in our study. First, our model was developed based on Chinese middle-aged and elderly men at a single-center, and the lack of an external validation cohort limits the universality of the model for application in other regions. Second, our study was an observational prospective cohort study; we controlled for numerous relevant confounders, but the possibility of residual confounding remains. Third, during follow-up, information about the dosage and duration of anti-osteoporosis drugs and other drugs that influence bone metabolism was not obtained, which might affect the evaluation of osteopenia risk.

## Conclusions

A nomogram and online web-based calculator for osteopenia in Chinese middle-old men was built and demonstrated good discrimination and calibration. By assessing individual risks, we can formulate effective interventions for patients and provide health education according to their lifestyles. This will be of great significance for the early prevention and management of middle-old men with high osteopenia risk.

## Data Availability

Data availability Some or all data sets generated during and/or analyzed during the present study are not publicly available due to privacy restrictions but are available from the corresponding author on reasonable request.

## Abbreviations

EHRs: Electronic Health Records
C-index: Concordance index
ROC: receiver operating characteristic curve
DCA: decision curve analysis
LNBMD: bone mineral density at left femoral neck
Hb: hemoglobin
ALB: serum albumin
PBG: postprandial blood glucose
FLD: fatty liver disease
WHO: World Health Organization
LBM: low bone mass
NOF: National Osteoporosis Foundation
OP: osteoporosis
IOF: International Osteoporosis Foundation
FRAX®: fracture risk assessment tool
BMD: bone mineral density
DXA: dual-energy X-ray absorptiometry
WC: waist circumference
RR: respiratory rate
HR: heart rate
BP: blood pressure
BMI: body mass index
FBG: fasting blood glucose
HbA1c: hemoglobin A1c
ALT: alanine aminotransferase
AST: aspartate aminotransferase
GGT: gamma-glutamyltransferase
Cr: serum creatinine
BUN: blood urea nitrogen
TG: triglyceride
TC: total cholesterol
HDL: high density lipoprotein cholesterol
LDL: low density lipoprotein cholesterol
PT: prothrombin time
APTT: activate part plasma prothrombin time
TT: prothrombin time
FIB: fibrinogen
TSH: thyroid stimulating hormone
TT3: total triiodothyronine
TT4: total thyroxine
FT3: free triiodothyronine
FT4: free thyroxine
LH: luteinizing hormone
FSH: follicle-stimulating hormone
T: testosterone
E2: estradiol
P: progesterone
OST: osteocalcin
P1NP: type I procollagen amino-terminal peptide
β-CTX: βisomer of C-terminal telopeptide of type I collagen
PTH: parathyroid hormone
25(OH)D: 25-hydroxy-vitamin D
ALP: alkaline phosphatase
CHD: coronary heart disease
CVD: cerebrovascular disease
CKD: chronic kidney disease
BPH: benign prostatic hyperplasia
IQR: interquartile range
AUC: areas under the ROC curve
OSTA: Osteoporosis Self-assessment Tool for Asians
NFκB: nuclear factor kappaB
TBS: trabecular bone score
Nlrp3: NLR Family Pyrin Domain Containing 3
Nrf2: nuclear factor erythroid 2­related factor 2
Keap1: Kelch­Like ECH­Associated Protein 1
NAFLD: nonalcoholic fatty liver disease
CSF1: colony-stimulating factor-1
TNF: tumor necrosis factor
c-Fms: colony-stimulating factor-1 receptor
MSCs: mesenchymal stem cells
